# Supplementary material for: Complexome profiling of the Chlamydomonas psb28 mutant reveals TEF5 as an early PSII assembly factor
Source: Plant Cell. 2025 Mar 17;37(6):koaf055. doi: 10.1093/plcell/koaf055 (PMC12164590; doi:10.1093/plcell/koaf055)
Supplement: koaf055_Supplementary_Data [file koaf055_supplementary_data.zip › psb28_interactive.zip!/plots/intensity/qualityPlots/peptides/MPPA2_Cre12.g509750.t1.2.html]

### Cre12.g509750.t1.2

Trivial name: MPPA2  
SeqCoverage [median]: 77.50  
Mass: 49.59 kDa  
  
 

| Sequence | MissedCleavage | Mass [Da] | wtIntensity | muIntensity | Score |
| --- | --- | --- | --- | --- | --- |
| AGKVVDALTAK | 1 | 1071.63 | 0.00 | 934.62 | 74.46 |
| AGRDHVAFGVDATR | 1 | 1470.73 | 24934.61 | 37234.72 | 99.93 |
| ALLDEARPTMPYQR | 1 | 1659.84 | 125931.80 | 117260.57 | 130.24 |
| ASVATAPGLAAAVGSSVLATGK | 0 | 1898.05 | 76607.15 | 20835.31 | 201.30 |
| DHVAFGVDATR | 0 | 1186.57 | 29451.68 | 30669.85 | 113.03 |
| DSLDAVK | 0 | 746.38 | 9284.19 | 5909.54 | 138.24 |
| DSLDAVKEQLAAQLR | 1 | 1655.88 | 31640.13 | 26427.02 | 234.90 |
| EHEVFTSVNPFAFAYK | 0 | 1884.90 | 4770.52 | 0.00 | 130.61 |
| ELEKIGATSFAR | 1 | 1320.70 | 2037.48 | 5957.76 | 79.49 |
| EQLAAQLR | 0 | 927.51 | 19450.75 | 12773.83 | 154.92 |
| EYVHSIMAPSR | 0 | 1288.62 | 20580.72 | 4726.40 | 88.19 |
| GGAGDIKSSAAASVVK | 1 | 1416.76 | 0.00 | 2418.61 | 82.59 |
| GTGLVGVVASGAPGK | 0 | 1268.71 | 105305.43 | 143710.98 | 284.10 |
| GVTDVQLATAK | 0 | 1101.60 | 134621.43 | 121113.86 | 270.54 |
| IGATSFAR | 0 | 821.44 | 119828.70 | 135621.34 | 182.13 |
| KEHEVFTSVNPFAFAYK | 1 | 2013.00 | 24530.35 | 0.00 | 139.58 |
| LNQLEALEILADAVVNAR | 0 | 1951.07 | 22286.80 | 0.00 | 155.92 |
| LPAVTEPPR | 0 | 978.55 | 51478.96 | 46995.06 | 141.39 |
| NMALGELR | 0 | 902.46 | 48644.70 | 43041.06 | 125.98 |
| NPLTAVNEVLHR | 0 | 1361.74 | 42837.70 | 81204.72 | 200.21 |
| SGGLLASVFGMGGGR | 0 | 1364.69 | 33542.65 | 0.00 | 163.90 |
| SGGLLASVFGMGGGRVEVPLSEK | 1 | 2246.17 | 1867.78 | 0.00 | 50.40 |
| SSAAASVVK | 0 | 818.45 | 69926.95 | 87577.82 | 171.75 |
| TAFEGGLGHSLVVDPSVVDGFTNETLK | 0 | 2788.39 | 5877.92 | 0.00 | 141.70 |
| TAPTFVTYGNLSSLPR | 0 | 1722.89 | 61150.17 | 35330.42 | 197.38 |
| TSTPATKPIVQTSSLR | 1 | 1685.93 | 172685.10 | 193840.94 | 173.03 |
| VASINTVSPISSLVLFVEGGAAAETPATAGASK | 0 | 3114.64 | 1348.62 | 0.00 | 71.71 |
| VDSIAKR | 1 | 787.46 | 600.53 | 2322.98 | 95.54 |
| VEVPLSEK | 0 | 899.50 | 28225.78 | 29320.20 | 136.11 |
| VEVPLSEKLPAVTEPPR | 1 | 1860.04 | 70646.77 | 100396.67 | 127.97 |
| VLEVAAFK | 0 | 875.51 | 120371.31 | 203867.44 | 139.31 |
| VVDALTAK | 0 | 815.48 | 33451.46 | 65572.77 | 116.73 |
| VVLAASGVDHAELTALATPLLNLHGNAHPAPQSR | 0 | 3439.83 | 21365.35 | 0.00 | 88.39 |
| YTYWEVR | 0 | 1015.48 | 15107.46 | 7163.02 | 131.04 |
|  |  |  | 1530391.0 | 1562227.5 |  |
